# Super-enhancers in immune system regulation: mechanisms, pathological reprogramming, and therapeutic opportunities

**DOI:** 10.3389/fimmu.2025.1652398

**Published:** 2025-08-15

**Authors:** Xin Lai, Caizhi Li, Xinglinzi Tang, Xinyi Luo, Feiyan Wu, Yuhong Liang, Bihui Huang, Hang Li

**Affiliations:** ^1^ The Seventh Clinical College of Guangzhou University of Chinese Medicine, Shenzhen, Guangdong, China; ^2^ Shenzhen Bao’an Chinese Medicine Hospital, Guangzhou University of Chinese Medicine, Shenzhen, Guangdong, China; ^3^ School of Pharmacy, Macau University of Science and Technology, Macau, China; ^4^ Scientific Research Center, The Seventh Affiliated Hospital of Sun Yat-Sen University, Shenzhen, Guangdong, China

**Keywords:** super enhancer, immune system, transcription regulation, cell signaling, pathogenesis

## Abstract

Super-enhancers (SEs) are dynamic chromatin structures that function as epigenetic hubs, orchestrating cell-type-specific transcriptional programs crucial for immune cell differentiation, functional specialization, and adaptive responses. These enhancer clusters integrate transcription factor (TF) networks, chromatin-modifying signals, and three-dimensional genome organization to govern lineage commitment, effector function acquisition, and metabolic reprogramming while enabling plasticity in response to environmental cues. SEs exhibit spatiotemporal regulatory properties, such as chromatin looping, phase-separated condensate formation, and stimulus-driven enhancer-promoter rewiring, all of which stabilize transcriptional outputs vital for immune homeostasis. Pathological dysregulation of SEs disrupts immune tolerance and amplifies aberrant transcriptional circuits, contributing to immune-mediated diseases marked by chronic inflammation, autoimmunity, or malignancy. Emerging therapeutic strategies targeting SE-associated components show promise in dismantling pathogenic enhancer networks through CRISPR-based editing, small-molecule inhibitors, and proteolysis-targeting chimeras(PROTACs). However, challenges remain in achieving therapeutic specificity amidst the dynamic reorganization of SEs and ensuring cell-type-selective delivery. By providing insights into SE-driven chromatin dynamics and transcriptional control in health and disease, this review focuses on two central questions: whether SEs causally drive immune cell fate decisions, and how they function within shared core transcriptional regulatory networks across cancer, infection, and autoimmune diseases. Future advances in multi-omics profiling, single-cell resolution analyses, and combinatorial therapeutic strategies will be critical for translating SE biology into precision interventions that restore immune equilibrium in dysregulated conditions.

## 1 Introduction

The transcriptional regulation of immune cell identity and function is governed by dynamic chromatin structures, with super-enhancers (SEs) acting as key coordinators of cell-type-specific transcriptional programs (1). SEs are rich in transcription factors (TFs), coactivators, and chromatin-modifying enzymes, functioning as epigenetic regulatory frameworks. They maintain cellular identity while enabling swift adaptation to environmental stimuli (2, 3). Within the immune system, SEs are integral to lineage commitment, effector function acquisition, and pathological reprogramming (1, 4). Epigenomic analyses have identified three critical properties of SEs essential for immune regulation: lineage-specific TF clustering, stimulus-responsive plasticity, and pathological permissiveness (1, 5). During hematopoiesis, SE-driven transcriptional hubs regulate immune cell identity by controlling master TFs such as BCL11B in T cells and PAX5 in B lymphocytes (6–8). This regulatory mechanism extends to innate immune cells, where pathogen sensing induces rapid remodeling of SEs at inflammatory loci through signal-dependent TF recruitment (9, 10). SEs function as epigenetic integrators of environmental signals, explaining how immune cells maintain a balance between the plasticity of differentiation and functional specialization (2, 3).

Despite these advances, two central questions remain unresolved in the field: whether SEs serve as causal drivers of immune cell fate decisions or merely accompany transcriptional changes as coactivators, and how SEs function within shared core transcriptional regulatory networks spanning cancer, infection, and autoimmune diseases.

The pathological consequences of SE dysregulation are increasingly recognized in immune-mediated diseases. Autoimmune disorders exhibit distinct patterns of SE aberrance at cytokine loci, while malignancies often exploit oncogenic SEs to sustain proliferative advantages (11–13). Comparative epigenomic analyses highlight both conserved and disease-specific SE architectures at key immune loci, suggesting context-dependent regulatory mechanisms (14). Recent advances in understanding SE biology have catalyzed novel therapeutic approaches, though challenges in target specificity and clinical implementation remain unresolved.

This review consolidates current knowledge of SE biology in immune regulation and critically examines ongoing debates. It first discusses the molecular foundations of SE organization and dynamics across immune cell lineages. Subsequently, it explores how SEs integrate developmental and environmental signals to coordinate immune responses. This review then evaluates the mechanisms underlying pathological SE reprogramming in immune disorders and examines emerging therapeutic strategies. Throughout, it identifies unresolved questions and proposes integrative approaches to advance both basic research and clinical applications.

## 2 Features and classification of SEs

### 2.1 Structural characteristics of SEs

**Figure 1:**
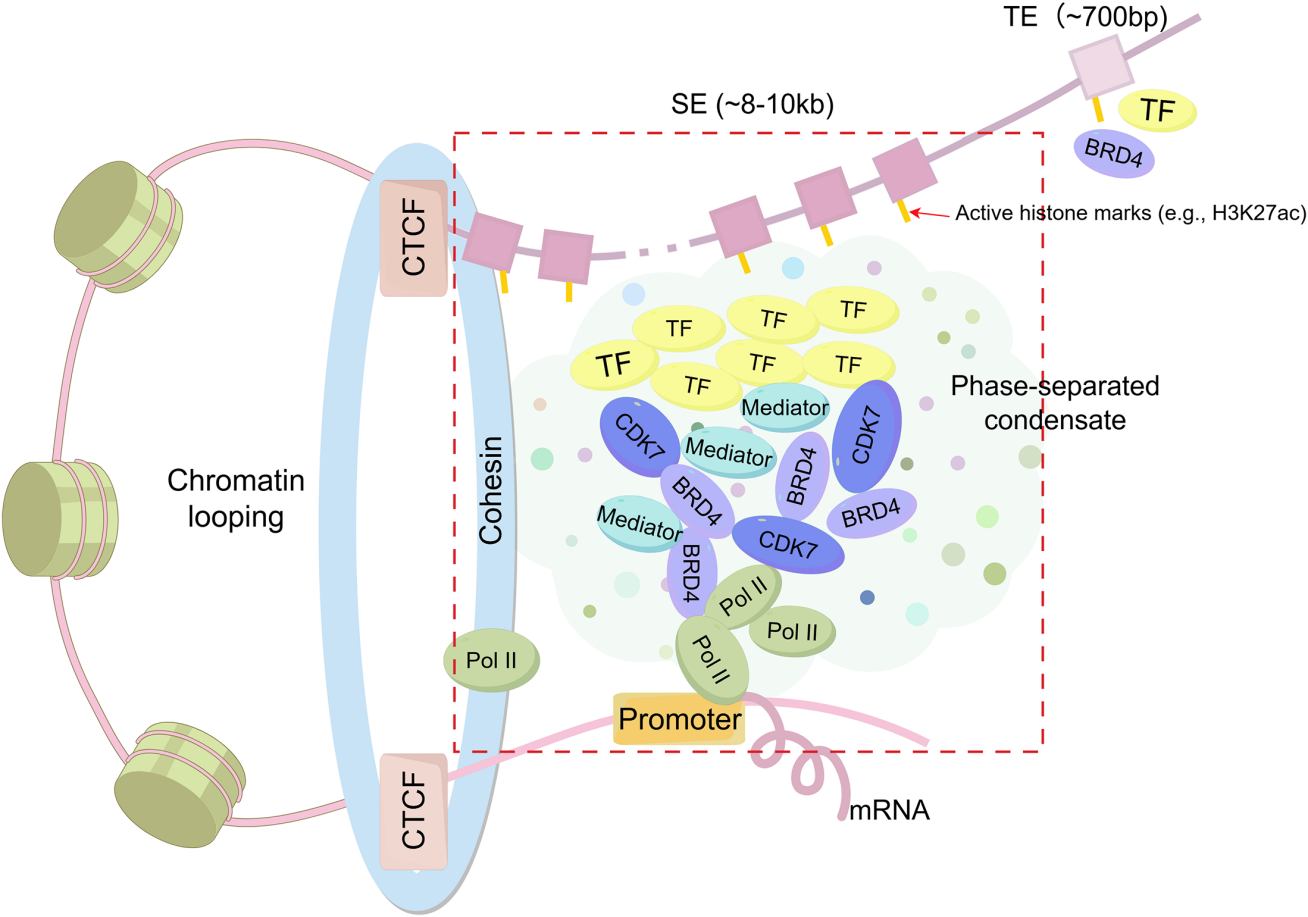
Structural features of Super-enhancers (SEs) compared to typical enhancers (TEs). SEs are large enhancer clusters (~8–10 kb) characterized by high-density binding of transcription factors (TFs), coactivators (e.g., BRD4, CDK7, Mediator), and RNA polymerase II (Pol II), which form phase-separated condensates in the vicinity of promoters. SEs are frequently associated with CCCTC-binding factor (CTCF)/cohesin-mediated chromatin looping. In contrast, TEs are smaller (~700 bp) and contain fewer gene transcriptional regulatory elements.

SEs constitute a distinct class of genomic regulatory regions, characterized by the clustering of enhancer elements that are densely enriched in TFs, coactivators, and other regulatory components (**Figure 1**). These specialized chromatin domains are characterized by unique epigenetic signatures, notably elevated levels of histone modifications such as H3K27ac and H3K4me1 (1).

The transcriptional activity of SEs is driven by three key architectural features: 1) A high-density aggregation of TF binding motifs, which enables the formation of autoregulatory circuits through cooperative interactions (15); 2) Enhanced chromatin accessibility, evidenced by a marked increase in H3K27ac deposition compared to typical enhancers (TEs) (16, 17); and 3) The synergistic integration of multiple SEs units, which collectively amplify transcriptional output through spatial coordination (1, 18).

Compared to TEs, which are relatively short (~700 bp) and less densely occupied, SEs are substantially larger clusters (~8–10 kb) composed of multiple enhancer elements that densely bind master TFs and coactivators such as Mediator (1, 4). SEs display markedly elevated levels of active histone modifications (e.g., H3K27ac up to 20–30-fold higher), generate abundant enhancer RNAs(eRNAs), and exhibit increased chromatin accessibility and DNase hypersensitivity (4). This biochemical and structural complexity underlies their stronger transcriptional activation potential, cell-type specificity, and sensitivity to perturbations.

Emerging evidence suggests that SEs utilize complex regulatory mechanisms that transcend conventional enhancer functions (12). These include liquid-liquid phase separation (LLPS) dynamics and cooperative TF assembly, both of which are vital for maintaining cellular differentiation programs through precise transcriptional regulation (19, 20). SEs not only amplify gene expression but also contribute to chromatin organization via LLPS, forming membraneless transcriptional condensates that concentrate TFs and coactivators (21, 22). This phase separation facilitates cooperative enhancer interactions, long-range chromatin contacts, and enhanced transcriptional output, thereby stabilizing transcriptional programs, particularly during dynamic processes such as immune cell differentiation and activation (21, 23, 24).

### 2.2 Classification and functional organization of SEs

An analysis of the SE landscape near the Wap locus during mammary gland differentiation revealed a temporal and functional hierarchy among its constituent elements (25, 26). Quantitative analysis of H3K27ac peak dynamics during cellular differentiation allows SEs to be categorized into three primary subtypes: conserved (Con), temporally hierarchical (TH), and *de novo* (DN) (**Table 1**). Con SEs are stable, evolutionarily conserved enhancers located at topologically-associated domains (TAD) boundaries, exhibiting stable chromatin interactions. TH SEs are dynamically regulated during differentiation, displaying stage-specific activation and chromatin remodeling. In contrast, DN SEs emerge at later developmental stages, often localize to focal interacting regulatory elements, and facilitate terminal gene expression via long-range chromatin interactions (14).

**Table 1:** Classification of SE subtypes.

Subtypes	H3K27ac Signal Dynamics	Features
Con	Persistently present	Stable gene expression; located in high gene density regions; conserved activity across cell types; enriched at TAD boundaries
TH	Established early with additional H3K27ac peaks acquired during differentiation	Moderately increased gene expression; less pronounced than DN; associated with TAD boundaries
DN	Gained in late differentiation stages or sporadically	Cell type–specific gene expression; located in low gene density regions

This table defines three SE subtypes based on their H3K27ac signal dynamics during cellular differentiation and their associated genomic and functional features.

## 3 SEs regulate immune cell differentiation and functional diversification

**Figure 2:**
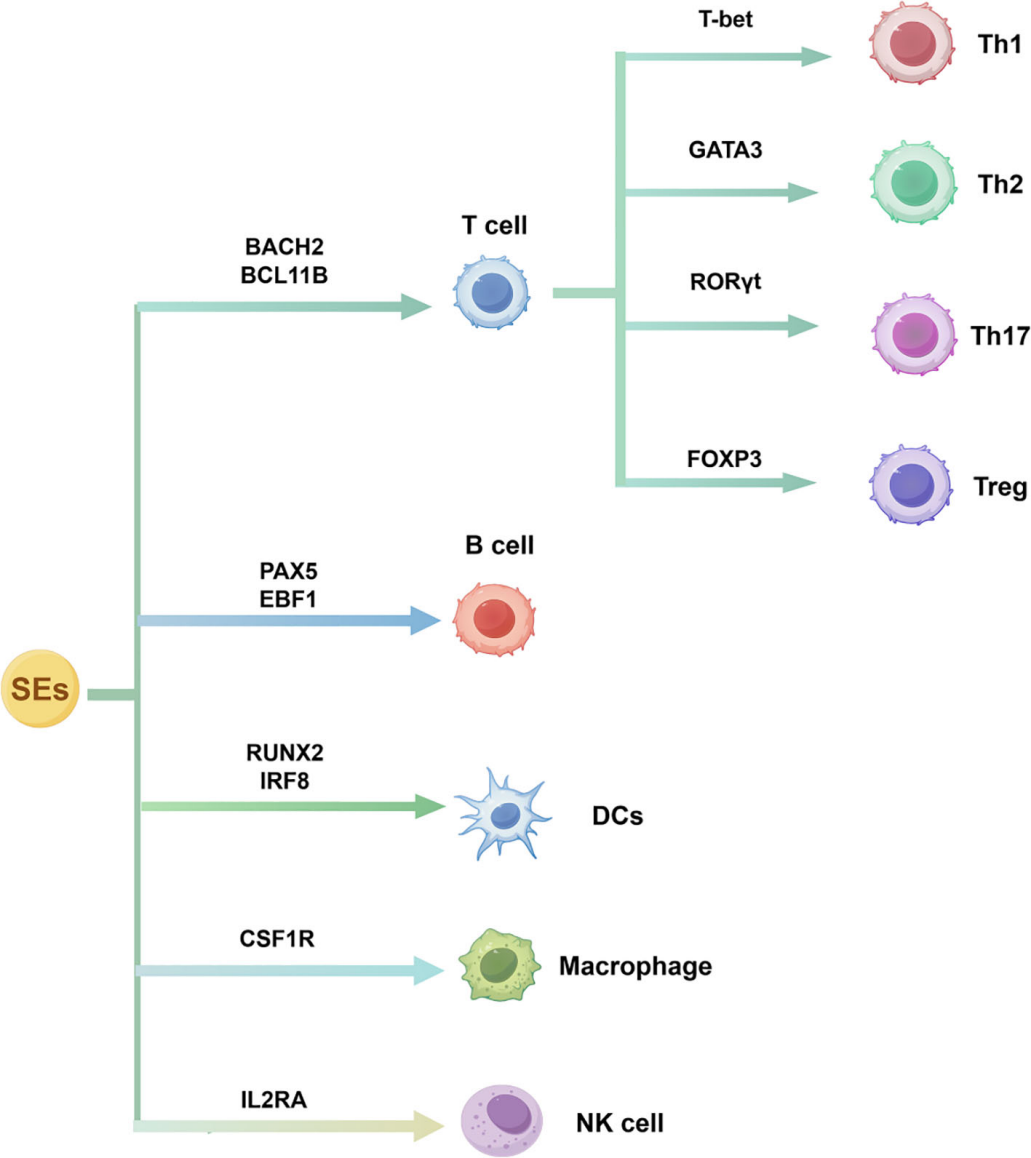
SE-driven transcriptional regulation of immune cell differentiation. SEs control lineage-specific TFs that govern the development of T cells, B cells, dendritic cells, macrophages, and natural killer cells, as well as T helper cell subset polarization.

SEs are complex three-dimensional chromatin structures formed by clustered enhancers that integrate chromatin-modifying signals and TF networks to regulate lineage-specific genes. Studies support the view that SEs may actively drive lineage commitment, rather than merely marking transcriptionally active regions (5, 27). They regulate immune responses through two key mechanisms: first, lineage-defining SEs stabilize the transcriptional machinery via chromatin looping (18); second, SEs mediate immune cell fate through epigenetic reprogramming (28). This chromatin plasticity provides a framework for understanding immune cell heterogeneity and differentiation. Moreover, dysregulation of SEs can contribute to pathological immune states, which will be discussed in detail in subsequent sections. The dynamic regulation of SEs in immune cells is shown in **Figure 2**.

### 3.1 SEs in T cell differentiation and function

SEs play a critical role in T cell differentiation, integrating signals from TFs, cytokine pathways, and chromatin remodeling to drive lineage commitment and functional specialization essential for adaptive immunity (1, 29, 30).

SEs regulate key cell fate genes like BACH2 and BCL11B through gene-specific chromatin architecture. For example, a SE at the BACH2 promoter maintains its expression via p300 and H3K27ac enrichment, while in BCL11B-dysregulated leukemia, a progenitor-specific SE ~730 kb downstream drives aberrant activation via chromatin looping, highlighting how SEs and genes are connected to shape immune cell identity and disease (6, 29). Upon activation, naive CD4+ T cells differentiate into distinct subsets such as Th1, Th2, Th17, and regulatory T cells (Tregs), each characterized by a unique epigenetic landscape stabilized by SE activity (29, 31). For instance, in Th1 cells, a TBX21 (T-bet) SE located within its gene locus drives lineage-specific expression and includes a distal ~146 kb enhancer cluster at the IFNG locus that enhances IFN-γ production. In Th17 cells, SEs linked to Rorc (encoding RORγt) are critical for IL-17 expression and directly suppress IL-4, together maintaining Th17 cell stability. Notably, recent studies demonstrated that deletion of this SE using CRISPR–Cas9 markedly suppresses Th17-mediated autoimmune responses, highlighting its therapeutic potential (see 2.5.1 CRISPR-Based Targeting of SEs in Immune-Related Diseases) (32).

Similarly, in Th2 cells, a downstream SE reorganizes TADs and chromatin loops to activate GATA3 is essential for establishing and maintaining their identity (33). In Tregs, a SE covering regions ~4kb upstream to ~12kb downstream of Foxp3 TSS promotes its expression for self-tolerance and Treg stability (31, 34, 35). These processes involve the recruitment of the mediator complex and chromatin-modifying enzymes, promoting enhancer-promoter looping and robust transcriptional activation of key effector genes.

SEs in T cells are closely linked to the expression of key genes, whose dysregulation is implicated in T-cell malignancies. In TAL1-positive T-cell acute lymphoblastic leukemia (T-ALL) cell lines, the TAL1 complex aberrantly activates SEs within the ARID5B locus, contributing to the development of T-ALL (36).

In summary, SEs serve as key regulatory hubs integrating TFs, cytokine signals, and chromatin remodeling to direct T cell lineage commitment and specialization. SEs at loci like BCL11B, TBX21, Rorc, GATA3, and Foxp3 highlight their essential role in cell fate control and their potential as therapeutic targets in T cell–mediated diseases.

### 3.2 SEs in B cell development and antibody production

During B cell differentiation, SEs dynamically reshape the chromatin landscape and integrate signals from lineage-specific TFs to coordinately activate key genes that define B cell identity (37, 38). The SE at the immunoglobulin heavy chain (IgH) locus comprises multiple B cell-specific enhancers that cooperatively silence germline transcription during V(D)J recombination (37). This SE is also induced in mature B cells to mediate somatic hypermutation (SHM) and class-switch recombination (CSR), both of which are essential for antibody diversification and antigen-specific responses.

In the early stages of differentiation, master regulators like PAX5 and EBF1 bind to SEs to activate B cell-specific genes and simultaneously repress enhancers linked to epithelial or stem cell-related genes, preventing lineage deviation (28, 38). SEs reorganize genome-wide in germinal centers to coordinate SHM and CSR via promoter-enhancer looping at the BCL6 locus, thereby maintaining a balance between antibody diversification and genomic integrity (37, 39).

Aberrant regulation of SEs is closely linked to B-cell malignancies. In chronic lymphocytic leukemia (CLL), DNA hypomethylation within SE regions leads to the abnormal activation of genes involved in lymphocyte proliferation and differentiation, while SEs associated with critical tumor suppressors (e.g., TP53) are lost, impairing terminal B cell differentiation (38, 40).

Together, these observations emphasize how SE homeostasis safeguards B cell differentiation, with perturbations potentially leading to aberrant lineage switching and cancer development (14, 38).

### 3.3 SEs in natural killer cells differentiation and dysregulation

The differentiation of NK cells is a complex biological process, characterized by distinct developmental stages and the precise coordination of cytokine signaling networks alongside transcriptional regulatory cascades (41–43). SEs in NK cells play a dual role: they orchestrate genome-wide transcriptional reprogramming while also regulating cellular activation, proliferative capacity, and effector functions (43).

Unlike T and B cells, SEs in NK cells are developmentally pre-programmed, maintaining functional stability independent of three-dimensional chromatin reorganization. This evolutionarily conserved regulatory framework enables the rapid deployment of cytotoxic responses against malignant and infected cells (44, 45). Furthermore, evidence suggests that the chromatin dynamics of SEs exhibit spatiotemporal regulation of NK cell activation, highlighting their central role in differentiation trajectories (46). Such spatiotemporal control is exemplified by the IL2RA SE, which recruits STAT5–MED1 complexes to fine-tune CD25 expression and integrate external cytokine signals into NK cell identity programs. Genome-wide profiling reveals that DNA hydroxymethylation can stabilize lineage-defining SEs and maintain their fidelity, thereby stabilizing the NK cell and innate lymphoid cell phenotype (47).

Within the immunoregulatory microenvironment, dynamic epigenetic modifications reshape SE accessibility, enabling SEs to integrate TF signaling, epigenetic remodeling, and Three-dimensional genome architecture to orchestrate NK cell development and function. For example, targeted epigenetic suppression of the β2-microglobulin SE can generate Human Leukocyte Antigen (HLA)-I-deficient effector cells that evade NK cell recognition while preserving immune compatibility (48). Moreover, chromatin-modifying enzymes, including the lysine demethylase UTX, regulate lineage-specific transcriptional programs through SE landscape remodeling, revealing novel therapeutic targets for immune modulation (49). UTX has been shown to modulate SEs associated with JUNB and IL2RB, which is crucial for NK cell identity (49). By advancing our understanding of these mechanisms, we gain deeper insight into innate immune cell developmental biology. While SEs play pivotal roles in NK cell differentiation under physiological conditions, their dysregulation in pathological contexts has been mechanistically linked to NK cell dysfunction. A notable example is found in natural killer T-cell lymphoma, where TOX2-SE-mediated chromatin looping brings enhancers and promoters into proximity, forming a feedforward circuit via RUNX3 recruitment that drives TOX2 overexpression and promotes lymphomagenesis (50).

SEs orchestrate NK cell differentiation and effector functions by establishing stable transcriptional programs and enabling rapid responses to external stimuli. Yet, the same regulatory architecture, when perturbed, can lead to disrupted NK cell activity and contribute to disease pathogenesis, such as lymphoma.

### 3.4 SEs in macrophage functional transitions

Macrophages are essential components of the innate immune system and remain central to studies on immune regulation. Toll-like receptor (TLR) 4 activation induces a distinct SE landscape in macrophages: SEs in activated macrophages are predominantly located near highly expressed genes, whereas genes associated with the resting state are positioned near repressed SEs and exhibit low expression following stimulation (51).

The epigenetic regulation of SEs is critical for monocyte-to-macrophage differentiation. Selective deletion of the CSF1R SE fms-intronic regulatory element in mice impairs CSF1R expression and tissue macrophage development in specific organs (e.g., brain, skin, kidney), while leaving other macrophage populations undisturbed, highlighting SEs’ tissue-specific role in lineage commitment and organ colonization (52). Palmitic acid treatment in human monocytes reshapes SE landscapes, enriching H3K27ac at SEs near inflammatory genes (e.g., IRAK2, IL6) and depleting it at SEs of phagocytosis-related genes (e.g., MERTK), linking SE dynamics to dysregulated inflammatory and homeostatic functions (53). Although BRD4 occupies SEs in macrophages, BRD4-deficient macrophages form functional alternative SEs, revealing compensatory mechanisms that maintain inflammatory gene expression (54).

Together, these findings underscore SE-mediated epigenetic reprogramming as a key driver of macrophage differentiation and plasticity, shaping functional transitions and highlighting therapeutic targets in complex diseases.

### 3.5 SEs in dendritic cells differentiation and pathology

DCs, as essential antigen-presenting cells in the immune system, originate from hematopoietic stem cells in the bone marrow (55). SEs play a critical role in regulating DC lineage specification by precisely modulating TF networks. Notably, experimental evidence demonstrates that in plasmacytoid DCs (pDCs), RUNX2-associated SEs coordinate cellular differentiation, maturation, and migratory capacity by enhancing RUNX2 transcriptional activity (56). Similarly, the IRF8 SE regulates the functional diversification of conventional DC subtypes (cDC1 and cDC2) through complex epigenetic mechanisms, thereby defining their unique immunological roles (57–59).

Dysregulation of SE architecture can lead to DC-mediated pathological conditions. A notable example is found in blastic plasmacytoid dendritic cell neoplasia, where RUNX2 SE drives malignant transformation by activating MYC and downregulating pDC lineage genes, thereby promoting leukemia cell proliferation and survival (56). Mutations in IKZF1 disrupt SE-mediated DC transcription by altering SEs that govern the expression of ID2 and BATF3, critical regulators of DC subset development and function, ultimately impairing IFN-α production and IL-12 secretion and resulting in defective DC subsets (60).

Overall, distinct SEs regulate different DC subsets by controlling specific transcriptional programs. The RUNX2 SE mainly drives plasmacytoid DC differentiation and migration, whereas the IRF8 SE directs cDC subtype specialization. Dysregulation of these SEs can lead to immune disorders, highlighting their importance in immune regulation and potential as therapeutic targets.

### 3.6 Other immune cells

Recent studies have further elucidated the role of SEs in various immune cell types, including less-studied myeloid cells like mast cells and neutrophils. GATA2 and MITF, key regulators of mast cell differentiation from myeloid progenitors, are controlled by SEs. Specifically, GATA2 promotes chromatin remodeling, enhancing SE activity and driving mast cell differentiation (61). Studies show that SEs in inflammatory clear cell renal cell carcinoma cells drive excessive CXC chemokine (e.g., CXCL1, CXCL8) production, altering neutrophil migration, survival, and gene expression. BET inhibitors suppress this SE-driven transcription, effectively limiting neutrophil changes (62). These findings illustrate that SEs regulate not only classic lymphoid lineages like T, B, and NK cells but also myeloid-derived immune cells. Moreover, they provide new perspectives for understanding the complexity of the immune system and offer potential targets for developing therapeutic strategies for specific immune diseases.

## 4 Mechanisms of SEs regulation of immune response

### 4.1 Regulation of cytokine expression

Emerging evidence indicates that SEs regulate immune cell development, differentiation, and inflammatory responses by dynamically reprogramming epigenetic modifications to modulate cytokine expression (2). In immune cells such as T helper cells (e.g., driving IFNG expression) and macrophages (e.g., IL-8 and IL-1β production), SEs collaborate with key TFs and form phase-separated condensates to drive the rapid and robust expression of cytokines and inflammatory genes during immune responses (29, 63). Studies have shown that eRNAs transcribed from the DNA sequences of individual enhancer elements within SEs can positively regulate the expression of protein-coding genes (11, 64). For instance, NF-κB binds to the IFNG locus, and the eRNAs transcribed from IFNG further associate with this complex to promote rapid IFNG expression in memory T cells. This mechanism enhances the expression of key genes, enabling memory T cells to exert their specialized functions and confer lifelong immunity, a fundamental aspect of adaptive immune responses (11, 65).

Dysregulation of SEs is implicated in various immune diseases. For instance, SEs modulate cytokine regulation by facilitating the binding of IRF4 and BATF3 to the Foxp3 SE, repressing its expression in Tregs (66). In autoimmune diseases like rheumatoid arthritis (RA) and systemic lupus erythematosus (SLE), inflammatory signals such as TNFα and IL-6 lead to epigenetic reprogramming of SEs, resulting in the aberrant overexpression of pro-inflammatory cytokines such as TNF, IL1 family, and chemokines (11, 67). Pathogenic SEs at the IL6 locus in RA drive inflammation through CD40–CD40L signaling (68). Moreover, defects in STAT1 signaling and subsequent SE reprogramming are closely linked to the pro-inflammatory phenotype of CD4+ T cells in spondyloarthritis, partly by upregulating IL1R1 expression (69). SEs represent critical regulatory nodes that coordinate epigenetic, transcriptional, and three-dimensional genomic networks in cytokine regulation.

### 4.2 Immune response signaling pathways

SEs act as epigenetic hubs controlling lineage specification in immune cells by forming extensive enhancer clusters that drive the expression of immunoregulatory master genes (5). SEs integrate both innate (e.g., TLR-NF-κB) and adaptive immune responses through dynamic interactions with key signaling pathways (10, 70).

In innate immunity, SEs amplify inflammatory responses by enhancing TLR-mediated transcriptional activation. Upon microbial recognition, TLR2 and TLR4 activate NF-κB and MAPK pathways, augmenting NF-κB-driven cytokine production, including pro-inflammatory TNF and context-dependent IL-10 (71–73). Similarly, IFN signaling promotes the formation of SEs at STAT1 and STAT4 loci through chromatin looping, while the rapid release of inflammatory cytokines such as IL-1β and TNF-α further amplifies the antiviral response (74). These mechanisms position SEs as critical transcriptional amplifiers in immune activation, regulating cytokine-driven lymphocyte differentiation, promoting Th17 polarization through IL17a/f, and impairing Th1 responses through TBX21 dissociation (75).

Studies have also uncovered complex cross-regulatory networks linking SE dynamics with cellular metabolic pathways. Upregulated CDK7 expression and activity enhance SE-driven AMPD3 transcription, leading to mTORC1 activation and metabolic imbalance (76). This metabolic imbalance facilitates the proliferation and survival of cystic cells, highlighting a mechanistic link between SE-driven transcriptional programs and metabolic reprogramming (76). Studies have identified a tumor-specific SE (PD-L1L2-SE) located between the PD-L1 and PD-L2 genes, which epigenetically co-regulates the expression of these two immune checkpoint molecules, thereby facilitating tumor immune evasion (77). This regulatory axis highlights SEs as central coordinators of immune microenvironment dynamics, influencing intercellular communication and therapeutic responsiveness (78, 79).

**Figure 3:**
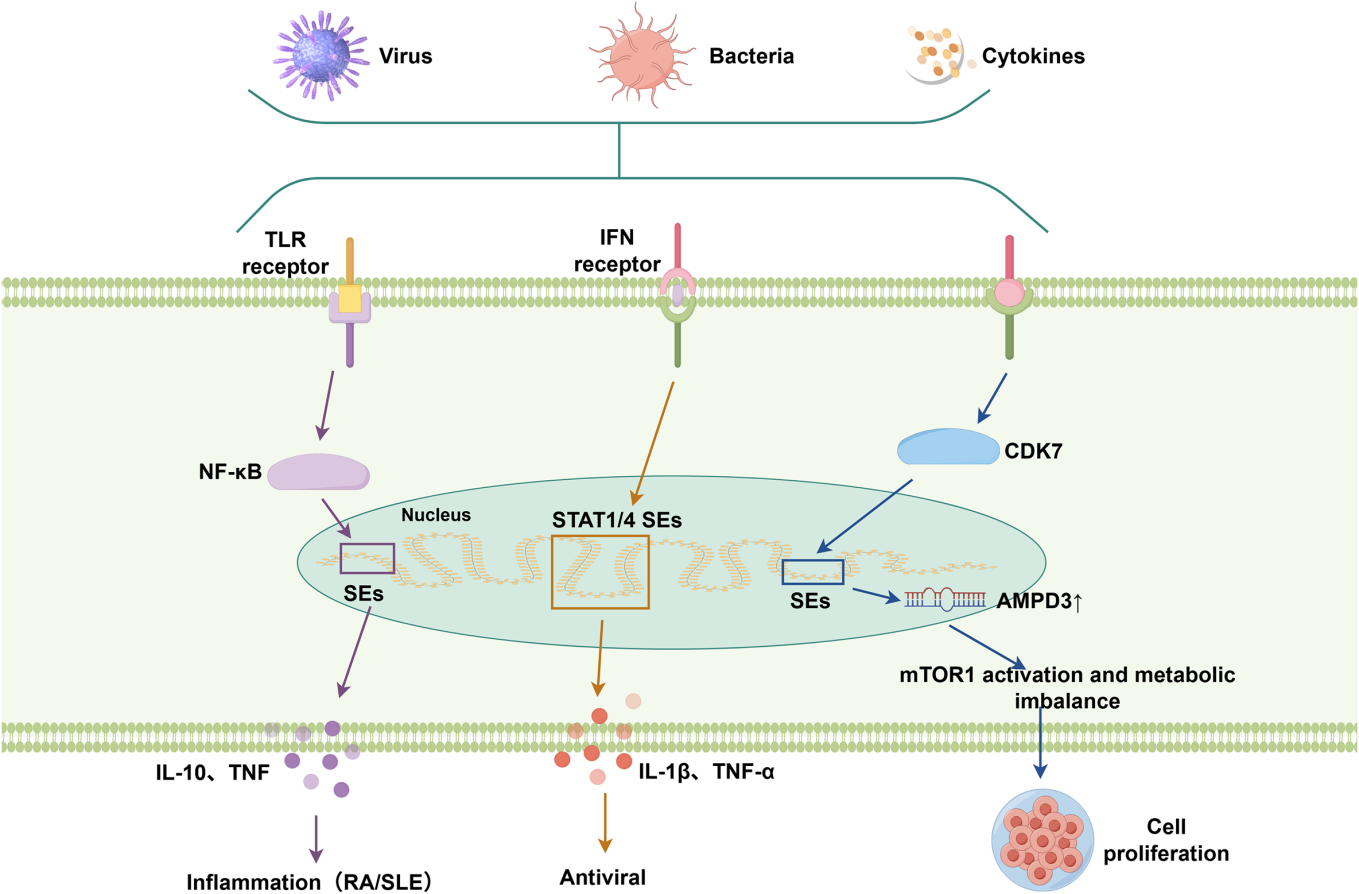
SE-associated signaling pathways involved in immune responses. SEs are activated in response to immune stimuli, including pathogens and cytokines. They integrate upstream signaling pathways (e.g., TLR, IFN pathways) via transcription factors such as NF-κB and STATs, and thereby modulate downstream pathways involved in inflammation, antiviral defense, and metabolic regulation.

SEs are integral to the regulation of immune responses, cellular differentiation, and metabolic pathways, underscoring their pivotal role in immune signaling and tumor microenvironment modulation (**Figure 3**). This highlights the necessity of precisely defining SE–gene networks within specific immune cell types to fully elucidate their context-dependent functions.

### 4.3 Epigenetic modulation of SEs in immune plasticity

By epigenetically controlling gene expression, SEs shape immune cell plasticity and subsequently affect cellular differentiation and functional states (5, 27). SEs are enriched with histone marks such as H3K27ac and H3K4me1, which correlate with transcriptional activity (1). Dynamic alterations in these modifications during immune diseases enable the rapid activation or repression of SE-associated immune genes. For instance, BET proteins recognize acetylated histones at SEs and recruit transcriptional machinery, amplifying cytokine production such as MCP-1 during acute inflammation, whereas BET inhibitors suppress SE activity and, through this mechanism, enhance antitumor immunity (77, 80).

DNA methylation at SE loci inversely correlates with enhancer activity, with hypomethylation destabilizing immune tolerance by derepressing pro-inflammatory genes in autoimmune diseases. Notably, in liver cancer, hypomethylation of the C/EBPβ SE further amplifies its own transcription and reshapes the enhancer landscape, thereby driving malignant phenotypes and tumor progression rather than directly impairing anti-tumor immunity (81–83). Targeting DNA methyltransferases or demethylases holds therapeutic potential for reprogramming SEs, while lncRNAs and eRNAs further modulate SE function by stabilizing chromatin looping or recruiting TFs, as exemplified by lncRNA-CSR interacting with IgH 3’RR SEs to regulate IgH recombination (84).

SEs also drive epigenetic reprogramming during immune cell transitions. In macrophages, SE reorganization upon lipopolysaccharide stimulation promotes polarization toward either pro-inflammatory (M1) or anti-inflammatory (M2) states (85). Similarly, in memory T cells, conserved SEs maintain accessibility to effector genes, enabling rapid recall responses (33). Disruption of these epigenetic programs impairs lineage-specific functions and immune surveillance.

## 5 SEs and immune-related diseases

### 5.1 Role in autoimmune diseases

**Figure 4:**
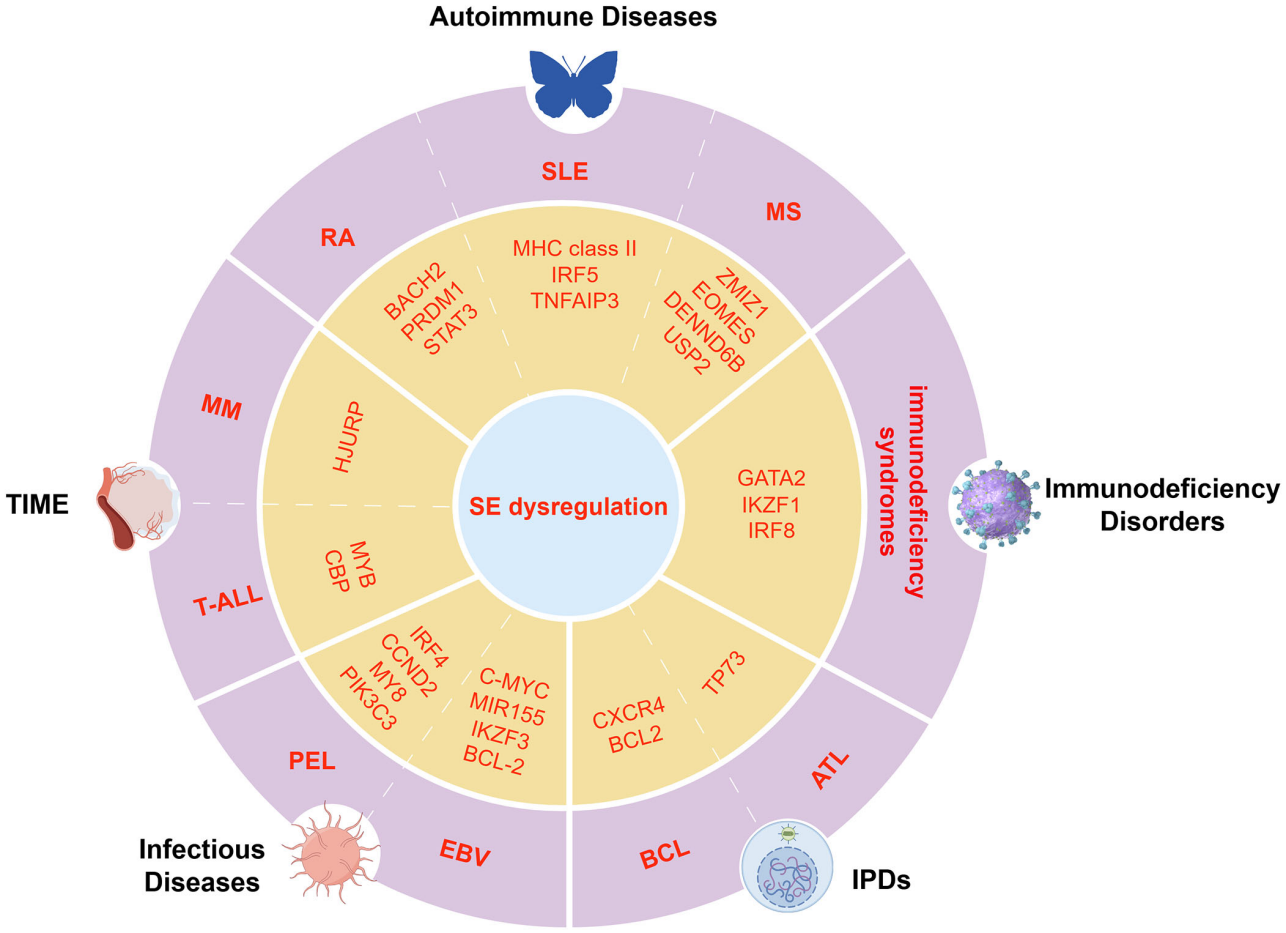
Dysregulation of key transcriptional programs mediated by SEs in immune-related diseases. Schematic illustration of disease-specific TFs and oncogenes regulated by SEs in immune-related conditions, including autoimmune diseases, immunodeficiencies, TIME, infections, and IPDs.

Autoimmune diseases such as RA, SLE, and multiple sclerosis (MS) result from a breakdown of immune tolerance, leading to chronic inflammation and tissue damage (11).SEs have emerged as critical regulators that amplify the transcription of immune-related genes involved in the pathogenesis of these diseases. In RA, disease-associated single-nucleotide polymorphisms (SNPs) preferentially localize within SE regions in CD4+ T cells, affecting the binding affinity of key TFs and coactivators, thereby dysregulating critical immune regulators such as BACH2, PRDM1, and STAT3 (11, 29) (**Figure 4**). Notably, PRDM1 and STAT3 cooperate to drive Treg differentiation and function through inflammation-dependent SE activity and TF network coupling, underscoring the critical role of interactions between SEs and genes in autoimmune dysregulation (86). This promotes sustained activation of inflammatory pathways, notably through NF-κB signaling and JAK/STAT signaling, thereby exacerbating cytokine production and joint inflammation (10, 21, 87).

In SLE, SEs are enriched near genes involved in antigen presentation and immune activation, including MHC class II, IRF5, and TNFAIP3, intensifying aberrant immune responses (88–91). In MS, the vitamin D receptor (VDR) binds 1,25(OH)_2_D_3_ to form SEs at risk loci such as ZMIZ1, EOMES, DENND6B, and USP2, where MS-associated SNPs (e.g., rs6589939) co-localize with PU.1 in open chromatin. These SNPs may disrupt VDR binding and CTCF-mediated looping, altering vitamin D-dependent immune pathways like phagocytosis and chemokine signaling. Vitamin D-dependent expression changes in monocytes further suggest that SE activity is modulated by environmental factors in MS (92).

RA, SLE, and MS show distinct SE dysregulation but share activation of inflammatory pathways and T cell dysfunction. RA involves BACH2/STAT3 SEs, SLE targets antigen presentation SEs, and MS involves VDR-related SEs influenced by genetics and environment. These highlight disease-specific regulatory mechanisms involving TFs and SEs that drive immune imbalance.

### 5.2 SE-mediated regulation of the tumor immune microenvironment

The TIME comprises diverse immune and stromal cells whose interplay determines tumor progression and response to therapy. Within this network, SEs epigenetically reshape tumor immune phenotypes by regulating key immune genes in cells like B cells, cooperating with factors such as CIITA to modulate chromatin, MHC II expression, immune checkpoints, and chemokine networks, thereby influencing antigen presentation, immune infiltration, and immunosuppression (93, 94).

In addition to immune modulation, SEs sustain oncogene transcription in diverse cancers. In hematological malignancies like T-ALL, TFs MYB and CBP cooperate to establish SEs that sustain oncogene expression (95, 96). Conversely, solid tumors such as gastric adenocarcinoma leverage SE-associated genes to epigenetically suppress CD8+ T cell cytotoxicity, thereby dampening antitumor immunity and reducing the efficacy of PD-1 checkpoint inhibitors (97, 98).

SEs frequently regulate key oncogenes, including MYC, via CTCF-mediated chromatin looping that ensures robust transcriptional activation (99, 100). In multiple myeloma (MM), SE-driven upregulation of HJURP fosters tumor cell proliferation and survival, while cooperating with SWI/SNF remodelers (e.g., me-BAF155) to suppress interferon pathways and weaken CD8+ T cell responses. Targeting this BRD4–me-BAF155–SE axis with CARM1 inhibitors can reprogram the epigenetic landscape and enhance antitumor immunity (101, 102). Moreover, SEs promote immune evasion by upregulating checkpoint genes like PD-L1 and driving inflammatory programs through TFs such as NF-κB, often in cooperation with SWI/SNF remodelers. This underscores their dual role in tumor progression and immunosuppressive niche formation, suggesting potential for therapeutic strategies integrating epigenetic modulation and immunotherapy.

SEs across cancers consistently drive oncogene expression and immune evasion. Hematologic cancers like T-ALL use SEs for oncogene activation, while solid tumors suppress CD8+ T cells via SEs. MM combines both to promote growth and immunosuppression, highlighting diverse SEs roles in tumor progression (103, 104).

### 5.3 Epigenetic regulation and SEs in immunodeficiency disorders

Immunodeficiencies encompass a range of disorders characterized by impaired immune function, often manifesting as increased susceptibility to infections, tumors, and autoimmunity (105). Studies have demonstrated that SEs play a central role in regulating the expression of key immune-related genes, and their dysregulation is closely associated with various immunodeficiency disorders. For example, disrupted SEs at the BACH2 locus reduce BACH2 protein levels by affecting protein stability, leading to misregulation of PRDM1 and abnormal B cell differentiation. This contributes to immune deficiency in BRIDA syndrome (106). This mechanism illustrates how heterozygous mutations affecting SE-regulated TFs like BACH2 can cause haploinsufficiency, ultimately manifesting as immunoglobulin deficiency, intestinal inflammation, and autoimmunity. Such pronounced dosage sensitivity is increasingly recognized as a common feature of SE-regulated genes, particularly those encoding lineage-defining TFs. Consistent with this, mutations within SE regions of other genes, including GATA2, IKZF1, and IRF8, have similarly been implicated in distinct immunodeficiency syndromes by disrupting the development and function of DCs, NK cells, and additional immune subsets (107, 108).

In addition, SEs are involved in the reprogramming of gene regulatory networks during viral infections, particularly in HIV. HIV-1 exploits SE-like elements within the host 5’ long terminal repeat to recruit multiple TFs, thereby promoting viral RNA transcription and sustaining latent infection (109). The virus preferentially integrates into host genomic regions proximal to SEs, and spatial repositioning of these regions during T cell activation may further increase the risk of integration and contribute to viral persistence (110). Host cells counteract this by employing epigenetic mechanisms, including DNA methylation, histone modification, and non-coding RNAs, to suppress SE activity and limit viral replication (111–114). The recently proposed concept of chromatin vaccines aims to reprogram enhancer-associated epigenetic states to boost antiviral immunity, and similar approaches may apply to SE regions given their pivotal role in immune regulation (109). Targeting SE-associated factors like BRD4 shows promise for restoring immune function and controlling pathogen activity in immunodeficiencies (115).

Immunodeficiencies involve SE dysregulation affecting key TFs like BACH2, GATA2, and IKZF1, leading to impaired immune cell development and function. Viral infections like HIV exploit SE-like elements to sustain latency, while host cells counteract by epigenetically suppressing SE activity. Together, these illustrate SEs’ central role in both immune deficiency and host defense across diverse contexts.

### 5.4 SEs in infectious diseases: immunomodulatory mechanisms and therapeutic implications

Recent studies have revealed that SEs play critical roles in the pathogenesis of infectious diseases. SEs not only regulate host immune responses to viral infections but are also involved in viral latency, replication, and the maintenance of virus-associated malignancies. In antiviral immunity, the RNA-binding protein SAFA enhances the expression of antiviral genes by activating SEs, thereby strengthening the host immune response (116). In HSV-1 and VSV infection models, SAFA deficiency impairs SE function, resulting in increased susceptibility and viral load in the host, highlighting the essential role of SEs in viral clearance and host defense (116). Viruses can also hijack host SEs to promote their own survival and replication. For instance, Kaposi’s sarcoma-associated herpesvirus utilizes its viral protein vIRF3 in cooperation with host TFs IRF4 and BATF to engage SEs, thereby regulating the expression of key survival genes such as IRF4, CCND2, MYB, and PIK3C3 in primary effusion lymphoma(PEL) and promoting tumor cell growth (117).

In Epstein-Barr virus (EBV) infection, SEs are co-opted by viral and host TFs to drive the expression of critical B cell survival genes such as c-MYC, MIR155, IKZF3, and BCL-2. EBV also interacts with host SE regions to modulate gene expression and regulate the switch between latent and lytic phases (118, 119). Notably, CRISPR-mediated disruption of specific SEs can induce lytic gene activation, highlighting their essential role in maintaining viral latency (118). SEs play pivotal roles in infectious diseases by modulating host immunity and being exploited by viruses for their own benefit. As such, SEs represent promising targets for the development of novel anti-infective therapies.

SEs play dual roles in infectious diseases by promoting host antiviral gene expression, exemplified by SAFA in HSV-1 and VSV infections, while also being hijacked by viruses such as KSHV and EBV to sustain tumor growth and maintain latency. This highlights SEs as critical nodes balancing immune defense and viral persistence.

### 5.5 SEs in immunoproliferative disorders

Immunoproliferative diseases, such as B-cell lymphoma (BCL) and MM, are characterized by abnormal proliferation of immune cells. Dysregulated activation of SEs plays a pivotal role in disease progression through various mechanisms.

SEs are central to the pathogenesis and progression of lymphoproliferative disorders like MM, sustaining high-level expression of oncogenes such as MYC, IRF4, and PRDM1. Notably, Chromosomal rearrangements reposition MYC near immunoglobulin and plasma cell–specific SEs, leading to its aberrant high expression. These SEs recruit dense clusters of TFs and coactivators, particularly BRD4 and the Mediator complex. This recruitment promotes chromatin accessibility and enhances transcriptional elongation, thereby amplifying transcriptional activity and exacerbating disease progression (13, 120, 121). Specifically, in BCL, distinct patterns of SE dysregulation, driven by both genetic and epigenetic changes, have been identified. These mutations within SEs disrupt TF binding, leading to the aberrant expression of proto-oncogenes such as CXCR4 and BCL2. This disruption promotes tumor cell survival and proliferation by activating several oncogenic signaling pathways such as the BCL6-BLIMP1 regulatory axis, NR3C1-mediated apoptosis inhibition, and the CXCR4-EPOR-JAK2 signaling pathway (39).

Beyond BCL and MM, SEs are also implicated in other immunoproliferative diseases. In adult T-cell leukemia(ATL), a SE located near the TP73 promoter enhances its expression by recruiting TFs such as IRF4, NF-κB, which in turn increase the expression of other TFs, including BATF3, JUNB, and FOSL2. These factors cooperate to activate TP73-driven DNA damage response and proliferation genes, such as PIDD1 and the MCM family, promoting cell growth. Additionally, Exon 2–3 deletions in TP73 further activate mTORC1 and NF-κB pathways, thereby amplifying proliferation and tumor progression (122). Immunoproliferative diseases share SE-driven oncogene activation that promotes cell proliferation. MM relies on MYC activation via SEs and rearrangements; BCL involves SE mutations disrupting TF binding to activate oncogenic pathways; ATL shows SE-mediated TP73 upregulation. Together, these highlight diverse yet convergent SE mechanisms promoting tumor growth and survival in different lymphoid malignancies.

## 6 Therapeutic strategies targeting SEs

### 6.1 CRISPR-based targeting of SEs in immune-related diseases

The advent of CRISPR-based technologies has revolutionized genome and epigenome editing, providing unprecedented opportunities for detecting and therapeutically modulating SEs in immune-related disorders (123). By enabling locus-specific editing of enhancer regions, these tools allow direct manipulation of SE architecture and associated chromatin states.

CRISPR-Cas9 facilitates the precise deletion or disruption of TF binding motifs at SE loci through site-specific double-strand breaks (**Table 2**). Although this approach can introduce irreversible changes and potential off-target effects compared to epigenetic interference strategies. For instance, as described above, deletion of the Rorc SE by Cas9/sgRNA electroporation effectively suppressed Th17-driven autoimmunity, highlighting the potential of cell-type-specific SE editing (32). In contrast to genome cutting, CRISPR interference reversibly suppresses SE activity by guiding dCas9-KRAB-MeCP2 to specific loci, reducing H3K27ac marks and disrupting SE-promoter looping. This blocks key TFs (e.g., IRF4, MEF2C), lowers oncogene expression (e.g., MYC, IRF4), and slows tumor cell proliferation, while minimizing risks to normal SE function (124). Conversely, CRISPR activation (CRISPRa) upregulates SE-driven gene expression by activating multiple enhancer elements, as shown at the SCREEM locus, where boosted eRNA transcription and H3K27ac marks enhance SNAI1 expression and promote EMT. Such locus-wide reprogramming illustrates how CRISPRa could tune immune cell phenotypes in a context-dependent manner (125).

**Table 2:** Comparison of Therapeutic Strategies Targeting SEs.

Category	CRISPR Editing	Small-Molecule Inhibitors	PROTACs
Mechanism	Precise SE or TF motif disruption	Inhibit SE-associated proteins (e.g., BRD4)	Degrade the components of the SEs
Advantages	precise, multiplexed, and adaptable modulation	Easy delivery, tunable and selective transcriptional control	Degrades hard-to-drug targets; Prolongs degradation, lowers dosing; Eliminates compensatory effects
Limitations	Off-target risk; delivery and safety concerns	Low specificity; transcriptional toxicity	Off-target toxicities; E3 ligase dependency

Although prime editing has not yet been applied to SEs, its high precision and double-strand-break-free mechanism present a future opportunity for fine-tuning enhancer elements or TF binding sites with minimal off-target effects (126).

Together, these CRISPR-mediated targeting of SEs enables precise, multiplexed, and adaptable modulation of regulatory networks through sgRNA-guided dCas9 variants, allowing functional dissection and therapeutic intervention in complex, polygenic gene control contexts (127). Future research should focus on cell-type- and environment-specific delivery systems, comprehensive safety profiling, and combinatorial strategies, including early or preventive interventions for autoimmune diseases. Integration with single-cell multi-omics and AI-driven modeling could help identify optimal SE targets and intervention windows, ensuring long-term therapeutic efficacy with minimal collateral effects on normal SE functions.

### 6.2 Small molecule-based therapeutic strategies targeting SEs in immune-related diseases

Small molecules present distinct advantages in modulating SE activity by penetrating cellular and nuclear membranes and targeting core components of SE-associated transcriptional machinery, such as BET proteins, CDKs, and HDACs (128). By disrupting protein–protein interactions or inhibiting essential enzymatic activities, small molecules can effectively suppress SE-driven transcription. BET inhibitors (e.g., JQ1, I-BET762) destabilize SE-driven oncogene or cytokine transcription by preventing BRD4 from binding to acetylated histones, thereby disrupting Mediator and Pol II recruitment at SE regions (129). In autoimmune models, these inhibitors reduce immune cell responses to pathogens by downregulating IFN-γ expression, although this broad suppression may also affect normal immune functions (129). CDK7/9 inhibitors (e.g., A51, A86) suppress SE-driven transcription by blocking phosphorylation of Pol II, thereby inhibiting transcriptional elongation. These agents have demonstrated significant antitumor activity in breast cancer and neuroblastoma models by targeting SE-regulated oncogenes such as MYC or MCL1 (130, 131). HDAC inhibitors induce histone hyperacetylation, selectively suppressing SE-driven genes in CLL by disrupting BRD4-Pol II interactions, including B cell-specific TFs and survival pathways (132). Although SE inhibitors show potent antitumor activity both *in vitro* and *in vivo*, their toxicity remains a significant limitation, resulting from widespread transcriptional dysregulation due to non-specific interference with SEs (133, 134). To address this, cell-type-specific delivery strategies such as antibody-conjugated or polymer-based nanoparticles are under development. For example, hydrophobic polyesteramide nanoparticles co-delivering BET inhibitor JQ1 (targeting BRD4) and CDK7 inhibitor THZ1 have shown synergistic SE disruption in pancreatic ductal adenocarcinoma, suppressing oncogenic programs (e.g., SMAD3, HES1, EGFR) and reducing systemic toxicity (135). Integration with single-cell profiling and AI modeling could further optimize timing and specificity for early or preventive intervention.

Small molecules have the potential to offer tunable and selective transcriptional control by targeting epigenetic regulators like BRD4 and CDKs, modulating enhancer-promoter interactions while aiming to preserve normal gene expression (136). Future research directions include exploiting the phase separation mechanisms of SEs to disrupt oncogenic SE condensates and employing machine learning approaches to predict SE–drug interactions, ultimately enhancing precision and reducing toxicity (137, 138).

### 6.3 Proteolysis-targeting chimeras in targeting SEs

PROTACs represent a novel and versatile strategy for selectively degrading disease-driving proteins via the ubiquitin-proteasome system (139). Unlike conventional small-molecule inhibitors that merely block protein activity, PROTACs are heterobifunctional molecules that recruit an E3 ubiquitin ligase to a target protein. This promotes its ubiquitination and subsequent proteasomal degradation (139). This approach is particularly advantageous for targeting SEs, which are driven by master TFs such as BRD4, as well as chromatin regulators with non-enzymatic scaffolding roles, which are often resistant to conventional inhibitors (140).

In immune-related conditions such as autoimmunity and cancer, pathogenic SEs sustain dysregulated expression of immune genes like cytokines and checkpoint proteins. PROTACs targeting SE-associated factors, especially BET proteins (BRD2/3/4), have demonstrated promise in preclinical autoimmune and cancer models (141). For example, BET-directed PROTACs like GNE-987 degrade BRD4, disrupt SE architecture, and produce sustained suppression of oncogenic or inflammatory transcription, often with greater efficacy than conventional BET inhibitors (142).

Unlike traditional inhibitors, PROTACs bypass the need for active site engagement, allowing the degradation of structurally challenging or non-enzymatic targets (143, 144). Moreover, transient exposure can achieve sustained target degradation, reducing the frequency of dosing and minimizing potential toxicity (145). This degradation removes both canonical and neomorphic functions of target proteins, helping circumvent compensatory pathways triggered by conventional inhibitors (146).

Despite their therapeutic potential, PROTACs face limitations including limited availability of E3 ligase ligands for optimization, and potential off-target toxicities, which may hinder their clinical efficacy (147). Future research should optimize next-generation PROTACs to expand E3 ligase options, integrate cell-type-specific delivery systems, and leverage single-cell multiomics and AI tools to refine target selection and dosing, ultimately enhancing safety and efficacy for early or preventive intervention in immune disorders.

## 7 Discussion

Recent advances have underscored SEs as dynamic hubs coordinating the transcriptional programs that govern immune cell identity, functional plasticity, and disease susceptibility. This review consolidates emerging evidence that SEs integrate chromatin architecture, TF networks, and environmental cues to modulate immune responses across physiological and pathological contexts. Despite these insights, key challenges continue to hinder the full translation of SE biology into clinical interventions.

A major methodological bottleneck lies in our limited capacity to monitor SE dynamics within the three-dimensional genome in real time. Current techniques, including ChIP-seq and Hi-C, provide only static snapshots, often lacking the resolution to capture spatiotemporal chromatin interactions and enhancer–promoter looping events that fluctuate during immune activation or suppression (148–151). Addressing this gap will require the development of advanced imaging methods, real-time chromatin conformation capture, and single-molecule tracking strategies to delineate SE behavior at the cellular level.

Another unresolved question is the insufficient understanding of feedback regulatory mechanisms that maintain or disrupt SE function. Although numerous studies have mapped TF binding and chromatin marks at SE regions, the feedback loops linking SE activity with TF availability, epigenetic modifiers, and metabolic reprogramming remain incompletely defined. Deciphering these dynamic regulatory circuits, including potential positive and negative feedback, is crucial to understanding how SEs sustain robust yet adaptable transcriptional outputs in diverse immune microenvironments.

Furthermore, integrating AI with single-cell and spatial multi-omics holds tremendous promise for decoding SE heterogeneity and predicting functional states. The rapid evolution of single-cell epigenomic and spatial transcriptomic technologies enables high-resolution mapping of SE landscapes across immune cell subsets and disease states (152–154). AI-driven analytical pipelines can help integrate these datasets, identify context-dependent SEs, predict their regulatory targets, and uncover potential off-target effects of SE-targeting therapies. Such integrative approaches are expected to facilitate the rational design of precision strategies, including patient-specific biomarkers for immunotherapy response and optimized combination treatments that leverage epigenetic and immune modulation.

To maximize translational impact, future research must address several practical challenges: the development of cell-type- and disease-specific SE modulators; the improvement of delivery systems to achieve selective targeting while sparing normal SE function; and the careful assessment of long-term safety and resistance mechanisms. Advances in PROTACs, CRISPR-based epigenetic editing, and small molecule-based therapies illustrate the innovative strategies on the horizon. Combining these tools with multi-omics and AI frameworks could help fine-tune dosage, minimize off-target toxicity, and extend therapeutic windows.

In summary, SEs represent pivotal epigenetic orchestrators of immune homeostasis and pathology. Bridging current methodological and conceptual gaps, including definitively resolving whether SEs causally drive immune cell fate and elucidating their role in shared transcriptional networks across diseases, will require collaborative efforts across molecular biology, bioinformatics, and clinical immunology. By advancing real-time SE tracking, decoding feedback regulation, and harnessing AI-powered multi-omics integration, we can transform our understanding of SE dynamics into actionable interventions, paving the way for highly specific and effective immunotherapies that restore balance in dysregulated immune states.
